# Rare case of “bullet” thrombus in the inferior vena cava

**DOI:** 10.1016/j.jvscit.2025.102012

**Published:** 2025-10-15

**Authors:** Jiri Molacek, Jan Baxa, Karel Houdek, Jan Wirth

**Affiliations:** a Vascular Surgery Department, University Hospital in Pilsen, Pilsen, Czech Republic; b Faculty of Medicine in Pilsen, Charles University, Pilsen, Czech Republic; c Department of Imaging Methods, University Hospital in Pilsen, Pilsen, Czech Republic

**Keywords:** Calcified thrombus, Cavotomy, Thrombectomy, Inferior vena cava, Vascular surgery

## Abstract

A bullet thrombus, characterized as an isolated calcified thrombus predominantly localized at the confluence of the renal veins in the inferior vena cava, is a rare clinical finding more commonly seen in pediatric patients than adults. The aetiology of bullet thrombi remains unclear. This report describes a 44-year-old female diagnosed with a bullet thrombus in the inferior vena cava, detailing the challenges encountered and the surgical intervention. The presence of a calcified thrombus in this region is typically an incidental discovery and complicates therapeutic decision-making, as there are currently no established guidelines due to the condition’s rarity.

An isolated calcified thrombus localized mostly at the confluence of the renal veins is called a bullet thrombus.^1^ The occurrence of calcified “bullet” thrombus in the inferior vena cava (IVC) is exceedingly uncommon and typically an incidental finding. It can be identified through the use of ultrasound (US), computed tomography (CT) and x-ray imaging. This phenomenon is more frequently observed in pediatric patients than in adults. The etiology remains unclear. In childhood, this pathology may be associated with an abnormal finding of the IVC or with a hypercoagulable status. In adults, a significant risk factor is recurrent deep vein thrombosis (DVT). There is a paucity of literature data on this pathology, particularly in the context of adult patients, and there are no established guidelines for treatment. This paper presents the authors’ experiences of treating an adult patient diagnosed with a bullet thrombus in the IVC. Written consent was obtained from the patient for the publication of this case report.

## Case report

**Fig 1:**
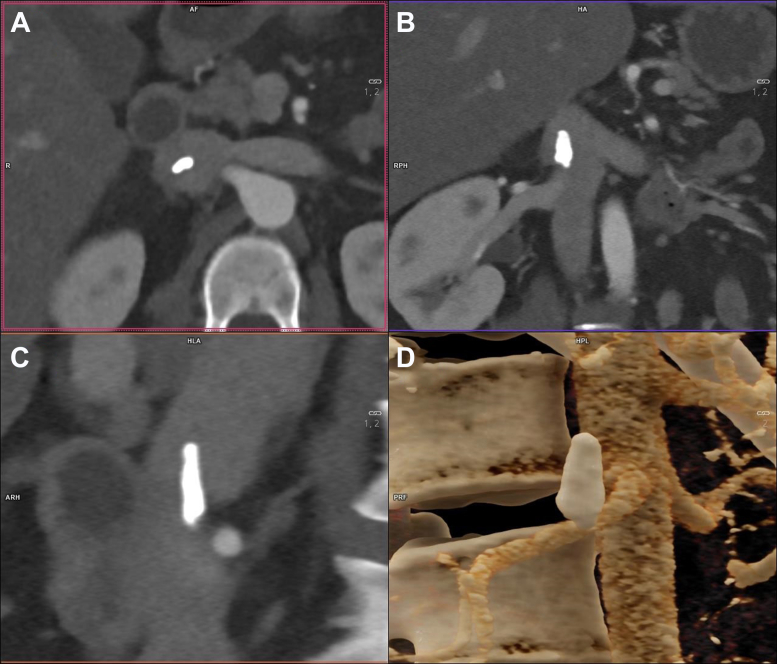
Computed tomograhy (CT) scans of bullet thrombus within the inferior vena cava (IVC) located below the right renal vein (axial **[A]**, coronal **[B]**, and sagittal **[C]** planes and corresponding three-dimensional reconstruction **[D]**).

A 44-year-old female was referred to our Vascular Surgery Department with an unusual finding within the IVC (just below the right renal vein). A contrast object is clearly visible on the CT scan (Fig 1). The patient has a history of recurrent DVT in the lower limbs over the past 2 decades, with one episode resulting in bilateral pulmonary embolism. DVT was localized only in the tibial vein and superficial femoral vein, not extending to the inguinal ligament. She was subjected to a comprehensive examination by an angiologist, who diagnosed her with an MTHFR mutation (mutation in a gene providing instructions for making an enzyme called methylenetetrahydrofolate reductase). The patient was using oral anticoagulation treatment with apixaban at a dosage of 10 mg per day. There is no history of any surgical or interventional procedures, including catheterization, drainage, puncture, etc. The atypical finding in the IVC was visible on US (contrast object not occluding the IVC, causing turbulent flow) and later on CT angiography. CT was indicated as an oncologic screening due to recurrent DVT during the most recent hospitalization for DVT.

The patient is currently asymptomatic. The multidisciplinary team’s consensus is that the object is a calcified bullet thrombus, and that a conservative approach would be the most appropriate treatment. Following a comprehensive discussion, the patient preferred surgical removal, having been fully informed about the risks associated with the procedure. Finally, this approach was accepted, and a operative solution seemed appropriate.

**Fig 2:**
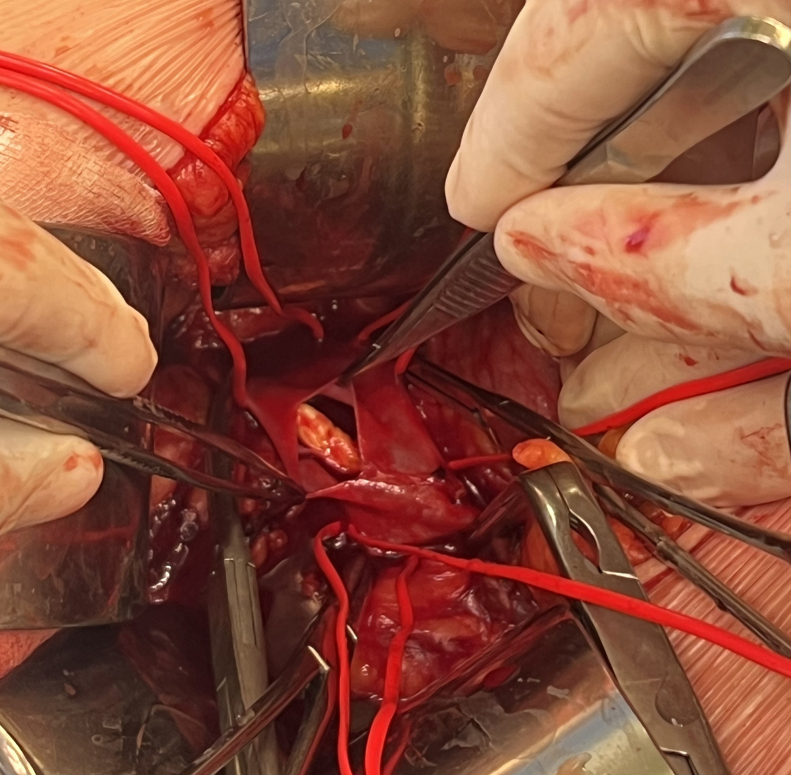
Exposed inferior vena cava (IVC) and performed cavotomy.

**Fig 3:**
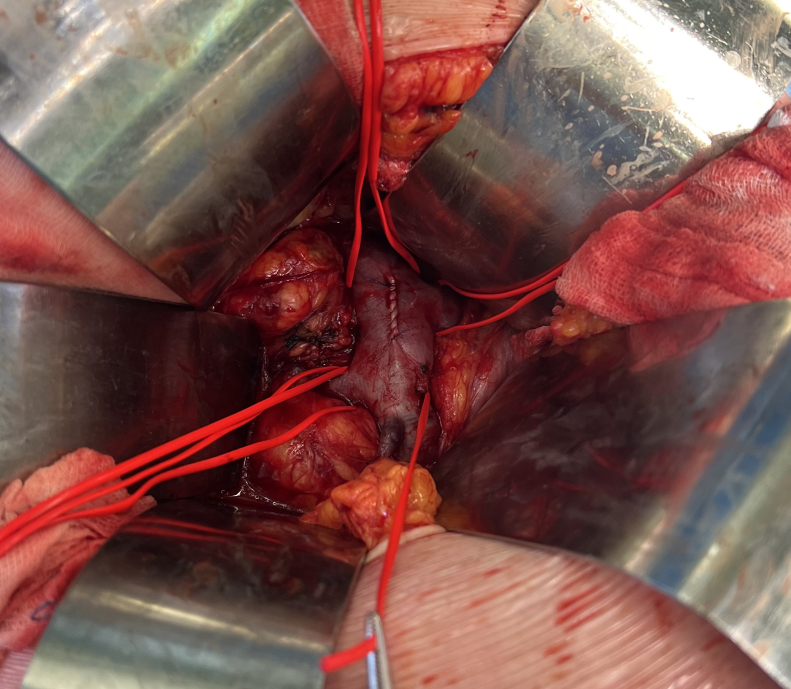
Sutured and declamped cavotomy.

Subcostal laparotomy was performed; exposure and looping of the entire pararenal segment of the IVC, as well as both renal veins, were done. Heparin was administered intravenously (7500 IU), and the IVC was clamped both centrally and peripherally, along with the renal veins. A longitudinal cavotomy was performed, allowing for the exposure and removal of the calcified thrombus (Fig 2). The thrombus adhered to the vein wall due to the presence of a small intraluminal flap or small valve. No histopathological analysis of the specimen was performed. Subsequently, the cavotomy was then sutured with a continuous 4/0 polypropylene suture, and a complete declamping was performed (Fig 3).

The postoperative period was uneventful, and the patient was discharged on the sixth postoperative day. The patient was managed postoperatively with low molecular weight heparin, changing for oral treatment (apixaban, 10 mg/day) on the third postoperative day. The patient is currently 6 months after the surgery and reports no problems at all concerning the procedure or thromboembolic disease.

## Discussion

The majority of thrombi observed in the IVC are associated with underlying malignancies, including renal, suprarenal carcinoma, and other cancer pathologies.^2^^,^^3^ This is a common finding that has been extensively documented in the literature, and the recommended guidelines for its management have been well-described. The literature on IVC bullet thrombi in newborns and infants is relatively extensive, whereas cases in adult patients are less well-documented.^4^, ^5^, ^6^, ^7^, ^8^, ^9^ The etiology of this condition in pediatric patients is associated with sepsis, dehydration, maternal diabetes, and other procoagulant states.^10^ The standard treatment approach for pediatric cases is conservative.^4^^,^^7^^,^^10^

In adult patients, bullet thrombus is a rare phenomenon, first described in 1961 by Singleton.^11^ The etiology remains uncertain. Congenital IVC disorders have been mentioned in the literature, as well as DVT and endothelial injury to the IVC.^12^ In the majority of cases, diagnosis of this pathology occurs incidentally during the course of an imaging procedure, such as an x-ray, US, CT scan, or magnetic resonance imaging. In the present case, the diagnosis of bullet thrombus was made during an oncologic screening procedure, subsequent to the occurrence of recurrent DVT.

In the majority of published cases, a conservative treatment was selected. A conservative approach was also considered in our case, but following a discussion with the patient, a surgical solution was selected. The decision to intervene therapeutically is not straightforward in the absence of symptoms. Following a thorough clinical examination and evaluation of potential future complications, in conjunction with the patient’s perspective and autonomy—carefully balanced against the ethical principles of nonmaleficence and beneficence—open surgery was determined to be the optimal treatment option. We did not find a case in the literature in which a bullet thrombus embolized and caused a fatal complication, but we could not completely exclude this situation. The 44-year-old mother of a young child was extremely anxious about the possible occurence of severe complications, particularly the recurrence of potentially fatal pulmonary embolism. We were aware that this major procedure has a significant morbidity and potential mortality; the use of an IVC filter was discussed, but due to the localization of the thrombus, it was decided not to use it. Following discharge, the patient will continue oral anticoagulant therapy, which is indicated lifelong due to recurrent thromboses.

## Conclusions

Calcified thrombus of the IVC is an extremely rare condition with no established treatment guidelines. Although the risk of embolization appears low, there remains a significant risk of subsequent thrombosis, due to alteration of haemodynamics of blood flow, as described by Virchow’s Triad. Management should be individualized based on thorough clinical assessment, risk evaluation, and patient preferences. Careful shared decision-making is essential to optimize outcomes.

## Funding

None.

## Disclosures

None.
